# The need for a refined classification system and national incident reporting system for health information technology-related incidents

**DOI:** 10.3389/fdgth.2024.1422396

**Published:** 2024-07-26

**Authors:** Md Shafiqur Rahman Jabin

**Affiliations:** ^1^ Department of Medicine & Optometry, Linnaeus University, Kalmar, Sweden; ^2^ Centre for Digital Innovations in Health and Social Care, University of Bradford, West Yorkshire, United Kingdom

**Keywords:** patient safety, healthcare quality, quality improvement, digital technologies, digital safety strategy

## 1 Introduction

Health information technology (HIT) has been defined as: “hardware or software that is used to electronically create, maintain, analyze, store, receive (information), or otherwise aid in the diagnosis, cure, mitigation, treatment or prevention of disease and that it is not an integral part of (1) an implantable device or (2) medical equipment” (1). Due to the immense advancement of HIT (interchangeably known as digital health, health informatics, information management, and technology in some health environments) in modern medicine, HIT incidents have been prevalent within healthcare settings over recent years (2). This is due to several reasons, such as asynchrony between the rapid growth of HIT systems and the complexity of the clinical workload, dysfunctional HIT systems, and inconsistency in the expectations of healthcare staff (3–5).

Despite many benefits, HIT systems pose sociotechnical complexities in healthcare and cause risks to patient safety, often in a new and unforeseen way (3–5). The challenge with digital health in relation to patient safety is the wide spectrum of digital health practices. These encompass instances of wrong diagnosis based on commercial names of medications or their physical attributes, a situation that can potentially lead to harmful health outcomes (6). Moreover, the rise of the complex volume of healthcare data and emerging artificial intelligence impose serious threats involving diagnosis and treatment recommendations, patient engagement, and administrative tasks. These complexities affect the entire healthcare organization, including patients, physicians, health workers, managers, and health systems cooperators (7). The use of social media, a vast and largely untapped data source, has been completely ignored in creating a classification system for HIT patient safety. This oversight highlights the urgent need for improved data sources, including social media, to enhance our understanding and management of patient safety in the digital health era (8).

Over time, a few classification systems have evolved in collecting, classifying, and analyzing patient safety HIT incident reports, including classification systems for medical devices that HIT may cover (9). Inconsistencies in these classification systems pose several challenges; for instance, HIT classification systems (HIT-CS) cover more concepts and classes than medical devices. The challenges also include a lack of classes for mitigating factors, actions taken to reduce risk, and ameliorating factors. This necessitates a greater need to improve existing HIT-CS to overcome these inconsistencies and challenges in regard to covered concepts and classes (9, 10).

At a population level, a national central system of reporting incidents increases opportunities to learn from mistakes by identifying hazards, risks, and opportunities to improve patient safety continuously (11). The rapid growth in the deployment of HIT systems and the prevalence of HIT incidents in modern healthcare demand a collaborative national direction towards improved safety of HIT systems aligning with future sustainability (12). This can be achieved by establishing a culture of a centralized national reporting system for HIT incidents, solely focusing on digital technologies as a new strategy (11, 12).

This opinion article highlights the urgent need for refined HIT-CS to remove the current complexities and establish a standard classification for patient safety concepts. We will also discuss how patient safety can be improved with the goal of a national reporting system for HIT incidents alone and why it should be prioritized.

## 2 The need for refining the existing HIT classification system

Millions of healthcare incidents are reported annually worldwide. For example, in NHS England alone, around 50,000 incidents per year are reported under the category of “medical device/equipment” (13). In Sweden, in 2020, around 1,000 incidents by healthcare providers and 5,000 incidents by manufacturers were reported under the “medical device” category to the Swedish Medical Product Agency (MPA), i.e., responsible for collecting medical device-related incidents and contributing to improved healthcare (14). However, refining HIT incidents from these medical device/equipment-related incidents is likely impractical since the numbers are still high. This may be due to the options being almost entirely physical devices, a few options for systems within a specific clinical context, or too many different digital systems to search by name (9, 15).

Incident classification is the process of categorizing incidents, adverse events, and near misses depending on the type, source, contributing factors, and symptoms with the help of applying standard taxonomy and terminologies (16). This helps in incident management accurately and consistently through the facilitation of communication, reporting, and analysis (17). The World Health Organization (WHO) Collaborating Centers for the Family of International Classifications (FIC) formed an international network of expert centers to fulfill the mission of health classifications, coding, and terminology development (18). The mission was aimed at improving health through the continuous development of health classifications and related products to retain their use and values globally. The WHO-FIC network promoted and focused on two WHO reference classifications, the International Statistical Classification of Diseases (ICD) (19) and the International Classification of Functioning, Disability, and Health (ICF) (20).

Jabin et al. emphasized in their study that the challenges of reporting incidents are inconsistency and lack of validation of the incident report classification (11). For example, the International Classification for Patient Safety (ICPS) was initially crafted to address the challenges in anesthesiology and later for the complete spectrum of the General Healthcare system. A further measure was taken to agree on a standard definition and preferred terms to develop the ICPS to set a global standard by the World Alliance for Patient Safety (21). However, the developed ICPS was still insufficient to classify the medical imaging-related incidents because the medical imaging department is more prone to health information technology (HIT) related challenges than any other department (11). For instance, the ICPS did not incorporate the issues related to HIT systems into the category of incident type. This necessitated us to embed the HIT system into the category of medical devices for the convenience of justifying the incidents.

Jabin et al., in their study (15), separated the HIT-related incidents from the general set of medical imaging incident reports and classified those incidents using the HIT Classification System (HIT-CS), proposed by Magrabi et al. (22). Although the ICPS and the HIT-CS complement each other, there are areas for improvement in both classification systems to a certain extent. For instance, the category of contributing factors and outcomes of the ICPS was identified to be all-inclusive compared to those of the HIT-CS (15). Moreover, the systematic review uncovered that most HIT-CSs comprised classes of concepts that were contributing factors/hazards and incident types. Like any other WHO patient safety classification system, there were no classes for mitigating factors, actions taken to reduce risk, ameliorating actions, organizational outcomes, detection, and incident/patient characteristics in these HIT-CSs. It is highly recommended that these concepts/classes be considered to enrich HIT-CSs (9). Moreover, contributing factors, such as human and technical factors, also need to be considered in the HIT classification system because of their common utilization (10, 15, 23). This implies that there is a continuous need for iterative refinement of existing frameworks, such as the ICPS and the HIT-CS, through extensive research and ongoing consultation with healthcare stakeholders (11).

Another way to refine these concepts is the application of thematic analysis for extracting information, which may not be possible using deductive analysis (e.g., ICPS, HIT-CS). As an illustration, when a database of thousands of incidents is analyzed, new concepts and themes emerge, which can ideally be added to the existing classification system, and incidents should then be iteratively coded (15, 24). This process should be supplemented by adequate training of healthcare staff before they are given the task of reporting incidents. This means there must be more detailed narrative texts of the incident reports before those incidents reach the investigation level (11, 25). This will require “international cooperation via the World Health Organization to develop an integrated framework incorporating systems that can accommodate information from all sources, manage and monitor things that go wrong, and allow the worldwide sharing of information and the dissemination of tools for the implementation of strategies which have been shown to work” (26).

## 3 The need for a national incident reporting system for HIT incidents

There are several challenges to the incident reporting system (IRS). For instance, one of the challenges regarding the IRS in Sweden is “decentralization.” This means each region in Sweden has established digital IRS, which are different from one another. These distinct digital IRSs include Synergi (Uppsala, Jonköping), LISA (Kalmar), and Platina (Halland, Gävleborg) (27). These varying systems add a layer of other complexities, such as interoperability among diverse systems and cross-regional comparisons hindering the continuous improvement in patient safety (11).

The Swedish MPA nationwide introduced reporting incidents related to medical devices, aiming to deliver, accord, and contribute to improved healthcare with the help of the Swedish eHealth Agency and the Swedish Authority for Privacy Protection (14). A similar stipulation has been set by the Medicines and Healthcare products Regulatory Agency (MHRA) in the UK to regulate and manage incidents with medical devices/products to address the issues regarding healthcare quality and patient safety (28). However, the challenge remains the same, i.e., refining HIT incidents from those associated with medical devices.

A unit-based reporting system is essential, focusing only on HIT incidents since each healthcare department comprises a busy environment and is unique in terms of the challenges it encounters (29). As an illustration, medical imaging has its own type of problem, unlike any other healthcare department, i.e., it is more susceptible to HIT-related issues than others. Moreover, a range of HIT systems are interconnected to different healthcare departments; therefore, a sole focus on HIT incident segregation should be prioritized at an early stage to ensure a sense of urgency to develop preventive and corrective strategies locally (11).

With this local development at each healthcare, we should also aim to develop a standard IRS at a national level. The national IRSs, such as MPA and MHRA, are responsible for checking those medical devices in Sweden and the UK, respectively, to comply with legal requirements; however, sole attention needs to be paid to digital technologies as a new digital safety strategy. Therefore, there is a need for a national IRS for HIT as a part of a comprehensive quality improvement initiative. This will not only reinforce knowledge of patient safety but also ensure safer systems through the implementation (6, 30).

Such an ideal system should have an independent organization to systematize digital health and patient safety surveillance, an agreed HIT classification system, a national repository for digital health information from all possible available sources, and a robust mechanism for setting priorities at local, national, and even international levels (if required). The system should be fair enough for the patient's rights, society, healthcare professionals, and facilities, which should be supplemented by an accountability process, right to anonymity, reporter's legal privilege, rapid feedback system, and a mechanism for effective dissemination by involving and informing all stakeholders (12, 31).

## 4 Discussion

Often, reporters and analysts concentrate on identifying and characterizing the incidents; however, they seldom focus on identifying the measures taken to improve the operations. A similar phenomenon has been noticed even when developing a framework or classification system. Managing the risks is more challenging than it seems because it involves the decision to implement an appropriate intervention, its implementation assessment, and its impact on the problem. On the other hand, disseminating preventive and corrective strategies in healthcare demands continuous communication and consultation among various healthcare professionals, managers, and patients (15, 32).

Actions taken to reduce risk, mitigating and ameliorating factors comprise steps taken to prevent the reoccurrence of the same or similar patient safety incident and improve system resilience. For instance, actions taken to reduce risk are those actions taken to reduce, manage or control the harm, or probability of harm associated with an incident (21). These actions may be either patient-related (e.g., provisional of adequate care), organizational-related (e.g., risk assessment), or both. Therefore, there is a need to establish a robust mechanism for including these categories and thus refining the existing HIT-CS on an ongoing basis.

Due to the different salient features of HIT incidents from medical device-related incidents, a unit-based reporting system at the local level is necessary. This will help focus solely on HIT incidents as each health department has its own type of challenges, which need to be addressed locally to develop preventive and corrective strategies applicable at the local level (33). This would eventually avoid any confusion among healthcare professionals in terms of the difference between HIT incidents and medical device-related incidents (6). For instance, in the UK, HIT incident regulations (mostly focused on the National Health Service, mHealth, Telemedicine, and Health data analytics) need healthcare services to report under the Health and Social Care Act 2008, General Data Protection Regulation and the Data Protection Act 2018.

The use of data to create a comprehensive and accurate classification system is crucial. However, the current system could be misleading as physicians and coders are not inclined to create additional work for them to add terms. It's important to note that patient safety and healthcare quality, although relatively new concepts are paramount. This underscores the urgent need for a more robust and inclusive classification system that can better serve our patients and improve healthcare outcomes and requires the collective effort and expertise of healthcare professionals, policymakers, and administrators (34). For instance, the feasibility of automatically generating hospital discharge summaries from inpatient records stored in electronic health records remains uncertain. The potential of these discharge notes as a data resource for a classification scheme on digital health and patient safety is immense, underscoring the value of our research and inviting further exploration (35).

Strengthening the quality standard of the IRS at the national level towards viable management to overcome the challenges faced at local and regional levels is one of the feasible means to resolve the current crisis of HIT incidents (12, 36). This call for action towards the “Digital Clinical Safety Strategy” should ensure that we use these digital systems safely and that these systems are designed and implemented so as not to harm patients. New digital clinical safety training materials, including optimized standards, guidelines, and best practice blueprints, can also supplement the strategy (12).

Despite several limitations of incident reporting, collecting information after it goes wrong is one of the most practical approaches, as most things that go wrong in daily clinical practice occur infrequently (15, 37). In general, systematic identification and characterization of HIT incident reports should be a high priority to improve day-to-day clinical practice (32). To achieve this agenda, appropriate steps should be taken in both dimensions—to ensure the safety of HIT systems and to employ those technologies as solutions to safety challenges. This can be achieved with the help of a standardized and comprehensive HIT-CS and cooperation across national directions towards a centralized IRS solely for HIT systems.
